# Pancreatic Beta-Cell Purification by Altering FAD and NAD(P)H Metabolism

**DOI:** 10.1155/2008/165360

**Published:** 2008-07-28

**Authors:** M. J. Smelt, M. M. Faas, B. J. de Haan, P. de Vos

**Affiliations:** Transplantation Biology and Immunoendocrinology, Section of Medical Biology, Department of Pathology and Laboratory Medicine, University Medical Centre Groningen, 9700 RB Groningen, The Netherlands

## Abstract

Isolation of primary beta cells from other cells within in the pancreatic islets is of importance for many fields of islet research. However, up to now, no satisfactory method has been developed that gained high numbers of viable beta cells, without considerable alpha-cell contamination. In this study, we investigated whether rat beta cells can be isolated from nonbeta endocrine cells by manipulating the flavin adenine dinucleotide (FAD) and nicotinamide-adenine dinucleotide phosphate (NAD(P)H) autofluorescence. Beta cells were isolated from dispersed islets by flow cytometry, based on their high FAD and NAD(P)H fluorescence. To improve beta cell yield and purity, the cellular FAD and NAD(P)H contents were altered by preincubation in culture media containing varying amounts of D-glucose and amino acids. Manipulation of the cellular FAD and NAD(P)H fluorescence improves beta cell yield and purity after sorting. This method is also a fast and reliable method to measure beta cell functional viability. A conceivable application is assessing beta cell viability before transplantation.

## 1. INTRODUCTION

Studies on primary beta cells are required in almost all fields of islet research. Unfortunately, it has been far from simple to obtain high numbers of pure and viable beta-cells. Previously published methods for purification of beta cells from other endocrine islet cells are based on the sedimentation velocity [1], labelling of beta-cell surface antigens [2], or the endogenous (auto) fluorescence of beta cells [3–6]. Most of these methods have a limited applicability, as they are associated with low yields of beta-cells, or permanent deterioration of cellular function caused by mandatory labelling of the cellular membrane.

A technology that we felt might be developed into an efficacious beta-cell purification method, with high yield, and without significant deterioration of function, is the sorting of beta cells based on their autofluorescence. Beta cells show higher autofluorescence than other islet cells. This autofluorescence is caused by the high cellular content of flavin adenine dinucleotide (FAD) [7–9], an electron acceptor in the metabolic oxidative phosphorylation. The principle applicability of isolation of beta cells by autofluorescence was shown by Rabinovitch et al. [4] and Van de Winkel et al. [10], but resulted in a preparation with considerable amounts of alpha-cells.

In this study, we investigated whether beta-cell yield and purity can be improved by manipulating islet cell metabolism and FAD autofluorescence. In addition, we investigated whether selection based on nicotinamide-adenine dinucleotide (phosphate) (NAD(P)H) fluorescence can be a valuable secondary isolation parameter. We show that preincubation of islet cells in culture media containing graded loads of D-glucose and amino acids alters cellular autofluorescence, beta-cell purity and yield after isolation.

## 2. MATERIALS AND METHODS

### 2.1. Experimental animals

Pathogen-free inbred male Lewis rats weighing 300–320 g were used as islet donors. All experimental animals were obtained from Harlan (Horst, The Netherlands). The animals were fed standard rat chow and acidified water ad libitum. All animal experiments were performed after receiving approval of the institutional Animal Care Committee of the Groningen University and all animals received human care in compliance with the Dutch Law on Experimental Animal Care.

### 2.2. Preparation of islet cells

Rat islets were isolated as previously described [11]. Islet isolation was performed in Krebs Ringer HEPES (KRH) buffered with 25 mmol/L HEPES containing 10% (wt/vol.) Bovine serum albumin (BSA). After ductal distention of the pancreas with KRH 10% BSA, the organ was chopped into pieces of 1-mm^2^. Next, the tissue fragments were subjected to 3 successive washing steps with KRH 10% BSA to remove proteases that might have leaked out of the exocrine part of the pancreas. Subsequently, the chopped pancreas was brought into a 25 mL Erlenmeyer flask and incubated at 37°C with 1.0 mg/mL of collagenase with KRH 10% BSA. The total volume of tissue and buffer was always 13 mL. After 10 minutes, the tissue fragments were placed in a 50 mL tube with 30 mL KRH 10% BSA at 4°C. The digested tissue was washed and sedimented 2 times to remove collagenase and exocrine-derived proteases. Next, the tissue fragments were placed again in a 25 mL Erlenmeyer flask and incubated at 37°C with 0.7 mg/mL of collagenase with KRH 10% BSA in a total volume of 13 mL. After 8 minutes, the tissue fragments were placed in a 50 mL tube with 30 mL KRH 10% BSA at 4°C. Finally, the digest was allowed to sediment and was washed twice with RPMI 1640 containing 1% (wt/vol.) BSA. Islets were separated from exocrine tissue by centrifugation over a discontinuous dextran gradient [12] and further purified by handpicking. Purified islets were washed in RPMI 1640 (Gibco) containing 1% BSA at least 8 times and cultured overnight in CMRL (Gibco) supplemented with 1375 mM D-glucose, penicillin, streptomycin, and 10% fetal calf serum (FCS) (Gibco, Calif, USA) at 37°C, 5% CO_2_.

The next morning, islets were collected and washed in phosphate-buffered saline twice before being dispersed into single cells by mechanical shaking at 37°C for 3 minutes in 0.05% trypsin, 0.5 mM EDTA (Gibco, Calif, USA). The enzymatic reaction was stopped by adding KRH 10% BSA. Cells were washed, and remaining cell clumps were removed by filtering the cell mixture over a 35 *μ*m pore size filter (BD Falcon).

On average, 550 islets per pancreas, and 1500 to 2000 cells per islet were obtained. Exclusion of trypane blue positive cells indicated that more than 95% of the cells were viable after the procedure.

### 2.3. Islet cell incubation

In the first experiment, dispersed islet cells were incubated in our standard medium, that is, RPMI 1640 (Gibco, Calif, USA) containing 1% BSA for one hour at 37°C before beta-cell sorting. Subsequently, in the second experiment, to study the influence of various concentrations of glucose or amino acids on beta-cell purity, dispersed islets were incubated in the following media containing different glucose and amino acids concentrations (see Tables 1(a) and 1(b)) MEM (Gibco, Calif, USA), RPMI 1640 (Gibco, Calif, USA), CMRL (Gibco, Calif, USA), or DMEM-F12 (Gibco, Calif, USA) all supplemented with 1% BSA for one hour at 37°C before cell sorting. In the third experiment, we evaluated the effects of glucose and amino acids on beta-cell purity separately; therefore, dispersed islets were incubated in unsupplemented MEM medium (5.5 mmol/L D-glucose and 553 mg/L amino acids), or MEM medium supplemented to either 10, 15, or 20 mmol/L D-Glucose (pH 7.4), or 1000, 1500, or 2000 mg/L amino acids (pH 7,4) for one hour at 37°C prior to flow cytometry.

In all experiments, twenty minutes prior to analysis 2 *μ*g/mL propidium iodide (PI) (Sigma-Aldrich, Mo, USA) was added to the cell mixtures to exclude apoptotic cells.

### 2.4. Flow cytometry and cell sorting

In experiments 1 and 2, high and low fluorescent cells were sorted on a fluorescence activated MoFlo high speed cell sorter (Dakocytomation BV, Heverlee, Belgium). In experiment 3, flow Cytometric analysis was performed on the LSR II system (BD Biosciences, Calif, USA). In all experiments, side-scattered light was collected at an angle of 90°, and signals were linearly amplified. The forward and side scatter patterns of the cells were analyzed at a wave length of 488 nm. The flavin adenine dinucleotide (FAD) content of the cells was analyzed at an excitation wave length of 488 nm, and collected at 525 nm. The PI staining was analyzed at an excitation wavelength of 488 nm, and collected at 675 nm. The nicotinamide-adenine dinucleotide (phosphate) (NAD(P)H) content was analyzed at an excitation wave length of 350 nm (UV) using an Argon laser, and collected at 450 nm. The 488 nm laser was operated at 80 mW and the Argon laser at 25 mW. The flow rate was typically a few hundred cells per second.

**Figure 1:**
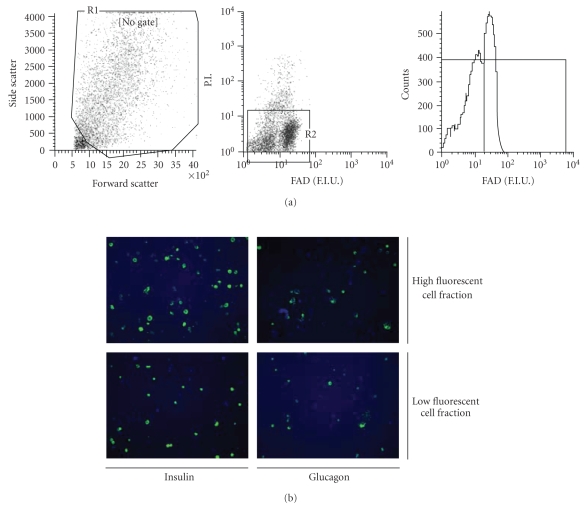
*Analysis of low and high fluorescent endocrine cells*. Forward and side scatter analysis of endocrine cells showed a very diverse cell population. Gating of propidium iodide negative cells showed two distinct, but overlapping cell populations when excited at 488 nm (a). Insulin and glucagon staining of sorted high and low fluorescent cell populations (b).

Flow cytometry results were analyzed using standardized gating on living cells using Winlist 5.0 software (Verity Software House, Topsham, Me, USA). After gating on the cell in the forward and side scatter plot, this gate was copied to a second plot in which we selected the living cells (i.e., propidium iodide negative cells) and both gates are then copied to a histogram showing FAD fluorescence (see Figure 1(a)). Analysis of the single cells by flow cytometry at an excitation wavelength of 488 nm resulted in two distinct autofluorescent cell populations. The first cell population showed low autofluorescence (7.24 ± 0.93 fluorescence intensity units (FIUs)). The second cell population showed high autofluorescence (29.94 ± 4.79 FIU) (see Figure 1(a)). In experiment 3, we measured the mean fluorescent intensity of the high and low fluorescent cells and evaluated the increase or decrease of fluorescent intensity (i.e., fold change) induced by glucose or amino acids versus unsupplemented medium.

### 2.5. Immunostaining

Both the high and low fluorescent isolated cell fractions were spotted on glass slides. Per slide, 50 000 cells were spotted, using a cytospin centrifuge. Cells were spun down at 500 rpm for 5 minutes at room temperature. Spots were dried, and the cells were fixed in Bouin fixative for 3 minutes at room temperature followed by immunostaining for the following pancreatic hormones or a combination of these hormones: insulin, glucagon, and somatostatin-pancreatic polypeptide (PP/somatostatin).

Briefly, slides stained for PP/somatostatin were blocked for endogenous peroxidase for 20 minutes in methanol containing 0.9% (vol./vol.) H_2_O_2_. Slides were washed and incubated with the primary antibody (polyclonal rabbit antirat PP (Abcam Inc., Mass, USA) 1:600, and monoclonal rabbit antirat somatostatin (Sigma-Aldrich, Mo, USA) 1:800) for 1 hour at room temperature. After washing in phosphate buffered saline, secondary (goat antirabbit IgG horse radish peroxidase (HRP) conjugated (Dako, Glostrup, Denmark) (1:50) in the presence of 1% goat serum) and tertiary antibodies (rabbit anti-goat IgG conjugated to HRP (Dako, Glostrup, Denmark) (1:50) in the presence of 1% Rabbit Serum) were incubated for 30 minutes at room temperature. The presence of an antibody-PP/somatostatin interaction was visualized after an enzymatic reaction using 3-amino-9-ethyl-carbazole (Sigma-Aldrich, Mo, USA). Cell nuclei were stained using haematoxylin for 1 to 3 minutes. Slides were embedded in Kaiser's glycerol gelatin (Merck & Co., Inc., NJ, USA). At random, at least 500 cells per spot were analyzed under light microscopy.

Slides stained for insulin or glucagon were incubated with the primary antibody (monoclonal mouse anti-rat insulin (IgG_1_) (Sigma-Aldrich, Mo, USA) 1:750; or monoclonal mouse anti-rat glucagon (IgG_1_) (Sigma-Aldrich, Mo, USA) 1:2000 for 1 hour at room temperature. After washing, the secondary antibody (goat anti-mouse IgG_1_ FITC conjugated (Dako, Glostrup, Denmark) (1:50) in the presence of 1% goat serum) was incubated for 30 minutes at room temperature in the dark. Cell nuclei were stained using 5 *μ*g/mL DAPI (Roche Inc., NJ, USA) for 20 minutes at room temperature in the dark. Spots were embedded in cytifluor (Agar Scientifics, Essex, England). The presence of insulin or glucagon staining was analyzed using a Leica DMRXA fluorescent microscope and Leica Qwin Pro Software. At random, at least 500 cells were analyzed per spot.

### 2.6. Statistics

Results are displayed as the mean ± standard error of the mean (SEM) of at least 4 experiments. The Mann-Whitney *U* test was used to determine statistically significant differences. *P*-values <.05 were considered to be statistically significant.

## 3. RESULTS

### 3.1. Experiment 1: fluorescent patterns in islet cells cultured in standard medium (RPMI 1640)

Isolated rat islets were dispersed into a single cell suspension by enzymatic digestion and incubated in RPMI 1640. On average, we obtained 1872 ± 507 viable cells (>95% of the total cell population) per islet (see Table 2). Analysis of the single cells by flow cytometry at an excitation wavelength of 488 nm [4, 10] resulted in two distinct autofluorescent cell populations. The first cell population showed low autofluorescence (7.24 ± 0.93 fluorescence intensity units (FIUs)), and comprised 46.25 ± 14.37% of the total viable endocrine cell population. The second cell population showed high autofluorescence (29.94 ± 4.79 FIU) and comprised 48.36 ± 6.96% of the total viable endocrine cell population (see Figure 1(a)).

In order to investigate the phenotype of these two distinct cell populations, the high and low fluorescent cell populations were sorted after which immunohistochemistry was performed. The low fluorescent endocrine cell population was composed of 13.52 ± 2.08% insulin positive beta cells (see Figure 1(b)) and 38.60 ± 12.95% glucagon positive alpha cells (see Figure 1(b)). The high autofluorescent cell population proved to be mainly beta cells (89.10 ± 5.45%) (see Figure 1(b)), but also contained a large portion of alpha-cells (18.65 ± 2.81%) (see Figure 1(b)).

These results show that beta-cell sorting based on cellular autofluorescence is possible; however, the purity needs to be improved.

### 3.2. Experiment 2: effect of preincubation with different media on beta-cell purity

As shown in the previous section, the vast majority of the beta cells were found in the high autofluorescent endocrine cell population. This population was, however, contaminated with a considerable number of glucagon-positive alpha-cells (18.65 ± 2.81%). We questioned whether we could change the fluorescent pattern of the high fluorescent beta-cell population and the low fluorescent nonbeta-cell population by altering the metabolic state of the endocrine cells.

**Figure 2:**
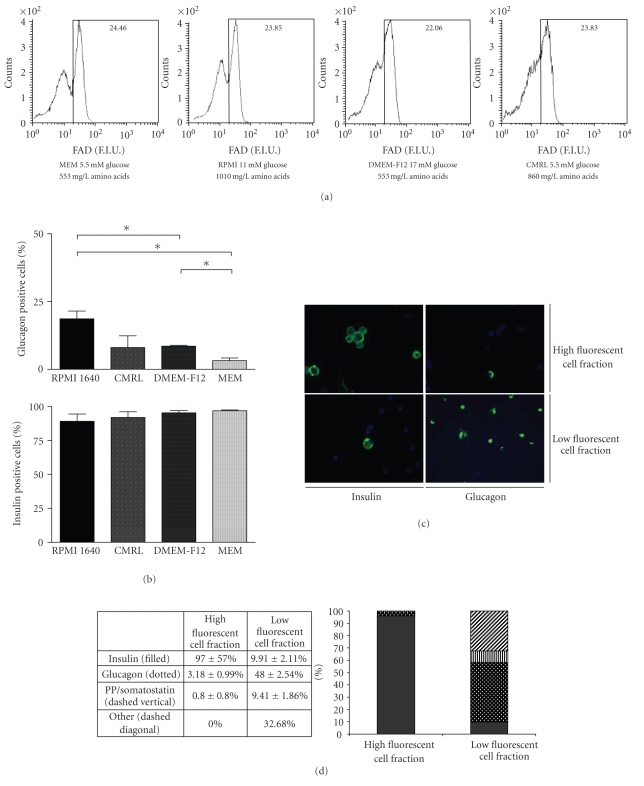
*FAD fluorescence is altered by culture medium composition*. Fluorescent patterns of dispersed islets preincubated in MEM, RPMI, CMRL, or DMEM-F12 medium (a). Analysis of glucagon-positive alpha cells present in the high fluorescent cell fraction following preincubation in MEM, RPMI, CMRL, or DMEM-F12 and subsequent sorting (b). Sorting of beta cells from the MEM incubated cell mixture resulted in a more than 95% pure beta-cell population, whereas in the nonbeta-cell fraction glucagon positive cells and a small amount of insulin positive cells were observed (c). Analysis of PP/somatostatin-positive cells present in the high and low fluorescent cell fractions following preincubation in MEM medium and subsequent sorting (d). Results are presented as the mean ± SEM, and statistical significance was determined using the Mann-Whitney *U* test. *P*-values <.05 were considered to be statistically significant and are represented by (∗).

The cellular metabolic state was altered in this second experiment by short-term incubation in other culture media, containing different amounts of D-glucose and amino acids. Both D-glucose and amino acids are well-known stimuli of metabolic activity and therefore suitable candidates to alter cellular flavin adenine dinucleotide (FAD) content and autofluorescence. To this end, endocrine single cells were incubated in RPMI 1640, MEM, DMEM-F12, and CMRL medium 1 hour prior to cell sorting. Table 1 shows the composition of the applied culture media. Preincubation of endocrine single cells in culture medium containing varying amounts of D-glucose and amino acids had a considerable effect on the FAD fluorescence of both the high and low autofluorescent cell population (see Figure 2(a)). When incubated in RPMI 1640, two partially overlapping fluorescent cell populations were observed. Preincubation of the cells in either DMEM-F12 or CMRL medium resulted in a more scattered fluorescence pattern in the low fluorescent cell population and a decreased difference in fluorescence intensity between the high fluorescent beta cells and the low fluorescent nonbeta-cell population (see Figure 2(a)). Despite the altered fluorescent pattern, sorting of the high FAD fluorescent cells still resulted in a considerable alpha-cell contamination. When dispersed islets were incubated in CMRL, alpha-cell contamination was not significantly different from preincubation in RPMI 1640. When cells were incubated in DMEM-F12 medium, alpha-cell contamination was reduced to 8.54 ± 0.36%, which was significantly lower compared to preincubation in RPMI 1640 (*P* = .03 Mann-Whitney *U* test) (see Figure 2(b)). However, preincubation of the endocrine single cells in MEM medium, containing only 5.5 mM D-glucose and 553 mg/L amino acids, showed two very distinct fluorescent cell populations with an increased difference in fluorescence intensity between the 2 populations compared to RPMI 1640 preincubation (see Figure 2(a)). Moreover, sorting of the high fluorescent beta-cell fraction resulted in only 3.18 ± 0.99% nonbeta-cell contamination (see Figures 2(b), 2(c), and 2(d)), which was significantly less than the nonbeta-cell contamination observed when the endocrine cells were preincubated in RPMI 1640 (*P* = .03 Mann-Whitney *U* test), and also DMEM-F12 (*P* = .03, Mann-Whitney *U* test) (see Figure 2(b)). The percentage of insulin positive cells in the high FAD fluorescent cell fraction slightly increased when incubated in either DMEM-F12 or MEM medium, but this increase was not statistically significant (see Figure 2(b)). Analysis of the presence of PP/Somatostatin positive cells in the high and low fluorescent cell fractions after preincubation in MEM medium and subsequent sorting showed the presence of these cells in the low FAD fluorescent cell fraction, but not the high fluorescent beta-cell fraction (see Figure 2(d)).

These data demonstrated that preincubation of endocrine single cells in culture medium composed of high or low concentrations of amino acids or D-glucose alters cellular FAD fluorescence. This altered FAD metabolism can be applied to increase the beta-cell purity by flow cytometry.

Both D-glucose levels and amino acids appear to influence beta and nonbeta-cell autofluorescence. Preincubation of single cell endocrine cells in MEM medium, containing both low concentrations of amino acids and D-glucose, resulted in higher beta-cell purity when compared to preincubation in standard RPMI 1640. However, preincubation in MEM still results in an alpha-cell contamination in the isolated beta-cell fraction.

### 3.3. Experiment 3: effects of glucose and amino acids on beta-cell autofluorescence

#### 3.3.1. Effects of glucose and amino acids on FAD fluorescence

As discussed in the previous section, preincubation of endocrine cells in MEM medium resulted in a beta-cell population of relatively high purity. However, the population still contained 3.18% alpha-cell contamination.

Both the D-glucose and amino acids differed in the varying media, and it is well known, and shown by the previous experiment, that both components influence the metabolic state, and thus the FAD content of the endocrine cells. Beta-cell purity and yield might be further optimized by preincubation in MEM medium supplemented with D-glucose or amino acids. Dispersed islets were incubated in unsupplemented MEM medium, or MEM medium supplemented with D-glucose to a concentration of 10, 15, and 20 mM, or amino acids to a concentration of 1000, 1500, or 2000 mg/L.

**Figure 3:**
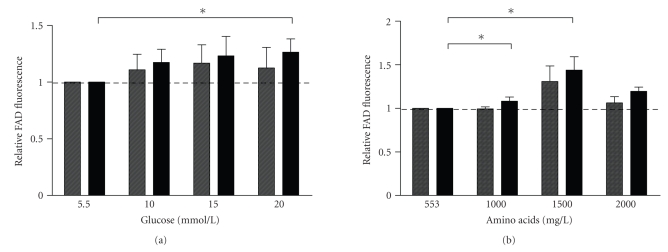
*Changes in cellular FAD fluorescence*. Beta-cell (black bars) and nonbeta-cell (dashed bars) FAD fluorescence were measured in response to (a) increasing concentrations of D-glucose and (b) amino acids. Fluorescence is represented as relative FAD fluorescence, compared to medium containing 553 mg/L amino acids and 5.5 mM D-Glucose. The graph represents four separate experiments, bars represent the mean ± SEM, and statistical significance was calculated using the Mann-Whitney *U* test. *P*-values <.05 were considered to be statistically significant and are represented by (∗).

Incubation of dispersed islets with increasing amounts of D-glucose showed an increased FAD fluorescence in the beta-cell population (high FAD fluorescent cell population), which was significantly increased at 20 mM D-glucose (FAD fluorescence fold induction of 1.26 ± 0.12 times when compared to unsupplemented MEM medium, *P* < .05, Mann-Whitney *U* test) (see Figure 3(a)). The FAD fluorescence of the nonbeta-cell population was not significantly influenced by incubation with increasing D-glucose concentrations (see Figure 3(a)). Incubation of single cell endocrine cells with increasing amounts of amino acids showed an increased FAD fluorescence in both the beta-cell and the nonbeta-cell population, where only the increased fluorescence of the beta-cell population was statistically significant (1500 mg/L: FAD fluorescence fold induction of 1.44 ± 0.15 times when compared to unsupplemented MEM medium, *P* < .05, Mann-Whitney *U* test) (see Figure 3(b)).

#### 3.3.2. Effects of glucose and amino acids on NADPH fluorescence

In addition to measuring FAD fluorescence, nicotinamide-adenine dinucleotide (phosphate) (NAD(P)H) fluorescence was also assessed as a secondary parameter to measure beta-cell metabolism by flow cytometry. Measuring autofluorescence at 350 nm represents both the cellular NADH and NADPH content.

**Figure 4:**
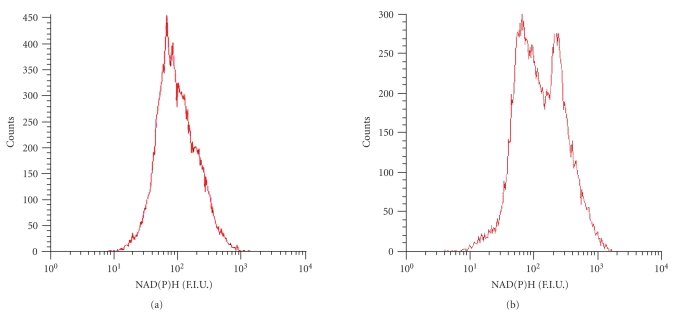
*Cellular NAD(P)H fluorescence*. NAD(P)H fluorescent pattern of dispersed islets following preincubation in (a) unsupplemented MEM medium and (b) MEM medium supplemented to 20 mM of D-Glucose.

Flow cytometric analysis of the endocrine single cell mixture incubated in unsupplemented MEM medium showed a single NAD(P)H fluorescent population when excited at 350 nm (see Figure 4(a)). When incubated in MEM with increasing amounts of amino acids, a more fluorescent cell population appeared as a minor shoulder (results not shown), whereas after incubation in MEM containing increasing amounts of D-glucose a highly fluorescent peak appeared (see Figure 4(b)). Thus, both glucose and amino acids appear to influence NAD(P)H fluorescence.

#### 3.3.3. The role of NADPH fluorescence in increasing the purity of the beta cells population using FAD fluorescence

**Figure 5:**
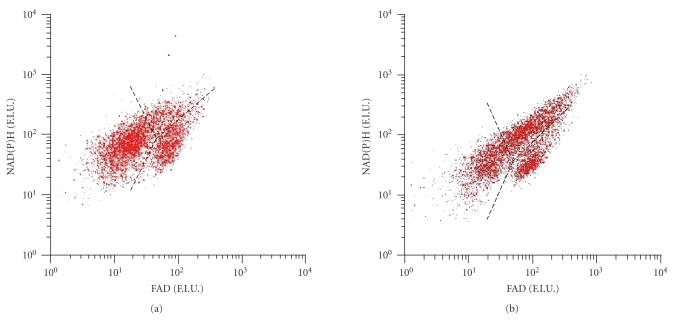
*Cellular FAD fluorescence versus NAD(P)H fluorescence*. FAD and NAD(P)H fluorescent patterns of dispersed islets following preincubation in (a) unsupplemented MEM medium and (b) MEM medium supplemented to 20 mM of D-Glucose.

Since both glucose and amino acids appeared to affect NAD(P)H fluorescence, the next step was to analyze whether it would be possible to use NAD(P)H fluorescence together with FAD fluorescence in order to get an even more purified beta-cell population compared with the use of only FAD fluorescence. Figure 5(a) shows that if cells are incubated in unsupplemented MEM medium, the dot plot of FAD fluorescence versus NAD(P)H fluorescence showed three populations: cells low in both FAD and low in NAD(P)H fluorescence, cells high in FAD fluorescence and low in NAD(P)H fluorescence, and a small population of cells high in both FAD and NAD(P)H fluorescence. When dispersed islets were incubated in MEM supplemented with D-glucose to 20 mmol/L D-glucose, the cell populations differ (see Figure 5(b)): the population of cells with high FAD, high NAD(P)H becomes much larger. Since in our first experiment we have shown that cells with high FAD fluorescent cells are beta cells and cells with low FAD fluorescent cells are nonbeta-cells, this may suggest that after stimulation with glucose, beta cells (cells high in FAD fluorescence) also become high in NAD(P)H fluorescence.

**Figure 6:**
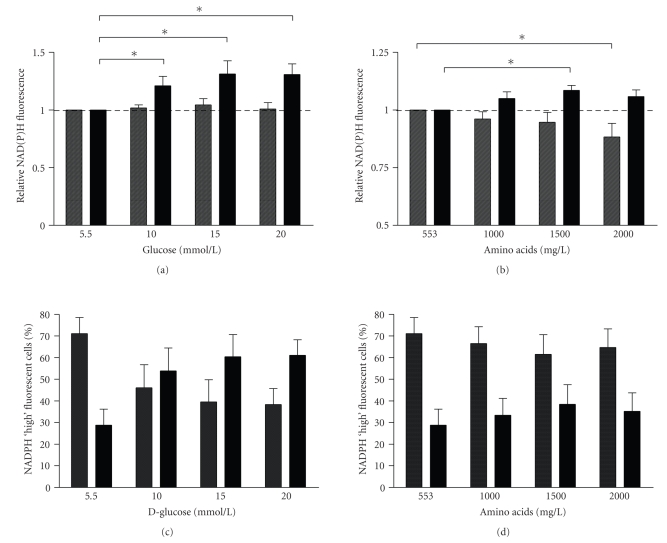
*Changes in cellular NAD(P)H fluorescence*. Beta-cell (black bars) and nonbeta-cell (dashed bars) NAD(P)H fluorescence were measured in response to (a) increasing concentrations of D-glucose and (b) amino acids. Fluorescence is represented as relative NAD(P)H fluorescence, compared to medium containing 553 mg/L amino acids and 5.5 mM D-Glucose. The percentage of high NAD(P)H fluorescent cells in response to (c) increasing concentrations of D-glucose and (d) amino acids was measured within the FAD fluorescent beta-cell population. Black bars represent the percentage of high NAD(P)H fluorescent cells within the FAD fluorescent beta-cell population. The dotted bars represent the low NAD(P)H fluorescent cells within the FAD fluorescent beta-cell population. The graphs represent four separate experiments, bars represent the mean ± SEM, and statistical significance was calculated using the Mann-Whitney *U* test. *P*-values <.05 were considered to be statistically significant and are represented by (∗).

In Figure 6 the effect of glucose and amino acids on NAD(P)H fluorescence of beta and nonbeta cells was analyzed. According the to results of experiment 1, beta cells were defined as cells high in FAD fluorescence, while nonbeta cells were defined as low in FAD fluorescence. Analysis of the NAD(P)H fluorescence level of these 2 cell population in dispersed islets showed that incubation in medium containing increasing amounts of D-glucose increased the NAD(P)H fluorescence in the high FAD fluorescent beta-cell population (1.21 ± 0.08, 1.31 ± 0.11, 1.31 ± 0.09 fold increase versus unsupplemented medium, respectively, for 10, 15, and 20 mmol/L D-glucose, *P* < .05, Mann-Whitney *U* test, see Figure 6(a)), whereas the low FAD fluorescent nonbeta-cell population was not influenced (see Figure 6(a)).

Incubation of dispersed islets in MEM supplemented with increasing amounts of amino acids showed a minor increase in NAD(P)H fluorescence in the high FAD fluorescent beta-cell population (1500 mg/L: 1.09 ± 0.02, *P* < .05, Mann-Whitney *U* test) (see Figure 6(b)). The low FAD fluorescent nonbeta-cell population showed a decrease in NAD(P)H fluorescence in response to increasing amounts of amino acids (see Figure 6(b)). This decrease was significant at an amino acid concentration of 2000 mg/L (0.88 ± 0.06, *P* < .05, Mann-Whitney *U* test).

Next, the percentage of NAD(P)H high fluorescent cells within the beta-cell (high FAD fluorescence) population was measured (see Figures 6(c), 6(d)). These figures show that approximately 71.2 ± 7.4% of beta cells were low NAD(P)H fluorescent cells, whereas 28.8 ± 7.4% of the beta cells were high NAD(P)H fluorescent when cells were incubated in unsupplemented MEM medium. When cells were incubated with MEM with increasing D-glucose concentrations (see Figure 6(c)), the percentage of high NAD(P)H fluorescent cells within the beta-cell population increased, while the number of low fluorescent NAD(P)H cells within the beta-cell population decreased (see Figure 6(c)). When supplemented to 20 mM D-glucose, the percentage of low NAD(P)H fluorescent beta cells decreased to 28.82 ± 7.41% (*P* = .06, Mann-Whitney *U* test), whereas the percentage of high NAD(P)H fluorescent beta cells increased to 61.12 ± 7.19% (*P* = .06, Mann-Whitney *U* test). After incubation with increasing amounts of amino acids, the percentage of high NAD(P)H fluorescent cells with the beta-cell population was unchanged (see Figure 6(d)).

Altogether, the results from experiment 3 show that D-glucose increased both beta-cell FAD fluorescence and beta-cell NAD(P)H fluorescence. This resulted in an increased percentage of highly NAD(P)H fluorescent cells within the beta-cell population after incubation with increasing D-glucose concentrations. Nonbeta-cell FAD fluorescence is only slightly increased after incubations with increasing D-glucose concentrations, while NAD(P)H fluorescence of the nonbeta cells is unaffected by glucose.

Amino acids slightly affect beta cells FAD and NAD(P)H fluorescence, but the percentage high/low NAD(P)H fluorescent cells within the beta-cell population remained unchanged. Nonbeta cells responded to incubations with increasing amino acid concentration by increasing FAD fluorescence and decreasing NAD(P)H fluorescence.

## 4. DISCUSSION

In this study, we show that both beta-cell and nonbeta-cell flavin adenine dinucleotide (FAD) and nicotinamide-adenine dinucleotide (phosphate) (NAD(P)H) fluorescence can be manipulated by preincubation of dispersed islet cells in culture medium containing graded loads of D-glucose and amino acids. The purity and yield of the sorted beta-cell fraction were optimized by incubation of dispersed islets in culture medium containing 5.5 mM of D-glucose and 553 mg/L amino acids. Under these conditions, the fluorescent pattern of the beta-cell and the nonbeta-cell fraction showed minimal overlap, resulting in a highly pure beta-cell fraction after sorting. Both beta-cell FAD and NAD(P)H fluorescence could be significantly increased by incubating dispersed islets in culture medium supplemented with 20 mM D-glucose. Nonbeta-cell FAD and NAD(P)H fluorescence were not affected by these conditions. Sorting of beta cells based on both the FAD and NAD(P)H fluorescence, after preincubation in culture medium supplemented with D-glucose, could provide a method to isolate a pure beta-cell fraction from rat islets.

The isolation method presented in this study clearly shows an improvement on the beta-cell isolation procedure described by Rabinovitch et al. [4] and Pipeleers and Pipeleers-Marichal [1]. When only FAD fluorescent sorting was applied, we could yield a beta-cell fraction with not more than approximately 3% alpha-cell contamination which is fivefold lower than the 16% contamination measured using the method described by Rabinovitch et al. [4] and Pipeleers and Pipeleers-Marichal [1]. The purity degree may be brought to 100% by applying an additional selection for NADP(H) fluorescence.

Our study shows a clear effect of the D-glucose content of the applied media on the FAD fluorescence and the ability to separate beta cells from nonbeta cells by flow cytometry. When the FAD fluorescence of beta cells cultured in MEM containing 5.5 mM is compared to MEM supplemented with 15 or 20 mM of D-glucose (with equal amino acid concentrations) (see Figure 3), we see a clear cut difference in the FAD fluorescence of beta and nonbeta cells. Although to a lesser extend, also amino acids influence FAD fluorescence of beta and nonbeta cells. However, the D-glucose and amino acid content of the culture medium are not the only factor influencing the cellular FAD fluorescence. This was shown in our second experiment in which we compared MEM medium to DMEM-F12 (with equal amino acid concentrations) and CMRL (with equal D-glucose concentrations) (see Figure 2). In this experiment, we found an effect of the medium on the FAD fluorescence of both cell populations, but it never reached the strong and distinguishable FAD fluorescence of beta and nonbeta cells as observed with supplemented MEM medium. These results may be explained by the differences in amino acid composition between the culture media. Different concentrations of amino acids (see Table 1(b)) that are known to stimulate beta cell insulin secretion, requiring oxidative metabolism, that is, L-Alanine, L-Glutamine, L-Arginine together with L-Leucine [13–18], may explain differences in FAD fluorescence as compared to supplemented MEM medium. But, as separate amino acids, concentrations are relatively low compared to earlier described stimulatory concentrations, the amino acids may work in synergy or DMEM-F12, and CMRL may contain other factors than D-glucose and amino acids that work in concert on the FAD fluorescence and cellular metabolism.

Our method of isolating beta cells on the basis of FAD and NADPH metabolism is not only applicable as a technique to purify beta cells but also as a fast and direct measure for the beta-cell functional viability. Cellular autofluorescence, measured at an excitation wave length of 488 nm, is dependent on the cellular FAD content. FAD is produced from FADH_2_ during ATP synthesis in the mitochondrial oxidative phosphorylation. Autofluorescence measured at an excitation wave length of 350 nm represents both the cellular NADH and NADPH content. NADH is the major electron acceptor during ATP synthesis. During active metabolism, FAD and NAD(P)H fluorescence are considered to be in redox equilibrium which implies that increases in FAD fluorescence and decreases in NADH fluorescence represent increases in cellular oxidative phosphorylation rates [19, 20]. Analysis of the beta-cell fraction showed no response in NAD(P)H fluorescence to increasing concentrations of amino acids, but a marked increase in NAD(P)H fluorescence when incubated with graded loads of D-glucose. These results suggest that besides a stimulation of ATP production and consequently the reduction of FADH_2_ to FAD and NADH to NAD^+^, other metabolic pathways are active leading to an increased cellular NAD(P)H content and fluorescence. It is well known that beta cells use alternative metabolic pathways, such as the pyruvate malate shuttle [21–27], to increase cellular NADPH levels in addition to the production of ATP. These pathways are considered to contribute to both the synthesis and exocytosis of insulin. When beta cells are stimulated with high concentrations of glucose, the pyruvate carboxylase activity is favored over pyruvate dehydrogenase, favoring both NADPH synthesis and ATP production. Therefore, measuring beta-cell FAD and NADPH responses to D-glucose can specifically be applied as a fast measure for the functional viability of the beta-cell preparation.

The NAD(P)H fluorescent patterns in the nonbeta-cell fraction suggest that NAD(P)H and FAD levels might not be fully in redox equilibrium in these cells. It might also represent a contamination of the nonbeta-cell fraction with beta-cells. Fluorescent staining of the nonbeta-cell fraction showed the presence of approximately 10% insulin positive beta-cells. The presence of these beta cells might skew the FAD/NAD(P)H equilibrium towards a more beta-cell like FAD/NAD(P)H balance. Measuring decreasing NAD(P)H levels in response to increasing concentrations of amino acids in the nonbeta-cell fraction confirmed the increase in FAD fluorescence, that is, the FAD/NAD(P)H equilibrium. The incubation of endocrine cells with increasing amounts of amino acids or D-glucose confirmed the finding that nonbeta-cell autofluorescence decreased in MEM which contains low concentrations of essential ingredients. The nonbeta cells appeared to have a stronger FAD response to increasing amounts of amino acids compared to the response to D-glucose, with an optimum at 1500 mg/L. In contrast to the FAD fluorescence, which showed an optimum at 1500 mg/L amino acids, the NAD(P)H decreased further when incubated with 2000 mg/L amino acids.

The described technology also allows for a clear identification and isolation of beta cells with different metabolic activity. Van Schravendijk et al. [28] have shown that different beta-cell subpopulations respond to either high or intermediate glucose concentrations, and these cells are well known as “high-” and “low”-responding beta-cells, respectively. With the combined application of FAD autofluorescence and NAD(P)H fluorescence, we show two distinct beta-cell populations. These two separate beta-cell populations might represent these “low”-responding beta cells and “high”-responding beta-cells.

The isolation approach described in this paper has advantages over earlier described beta-cell isolation methods. Isolation of beta cells based on the detection of the high beta-cell FAD and NAD(P)H fluorescence provides a tool to separate beta cells with minimal interference in cellular physiology. We showed that the yield and purity of the isolated beta-cell fraction could be optimized by preincubation of the dispersed islets in MEM medium supplemented with D-glucose. Under these preincubation conditions, both beta-cell FAD and NAD(P)H fluorescence were increased, whereas the nonbeta-cell fluorescence was virtually the same.

## Figures and Tables

**(a):** 

	D-Glucose (mM)	Amino acid content (mg/L)	FAD (mg/L)	Riboflavin (mg/L)
MEM	5.5	553	0	0.1
RPMI	11	1010	0	0.2
CMRL	5.5	860	1	0.01
DMEM-F12	17	553	0	0.219

**(b):** 

	L-Glutamine	L-Alanine	L-Leucine	L-Arginine-HCL
	mM (mg/L)	mM (mg/L)	mM (mg/L)	mM (mg/L)
MEM	0 (0)	0 (0)	0.4 (52.0)	0.6 (126)
RPMI	2.0 (300)	0 (0)	0.4 (50)	1.1 (240)
CMRL	0 (0)	0.3 (25)	0.5 (60)	0.3 (70)
DMEM-F12	2.5 (365)	0.05 (4.45)	0.5 (59.05)	0.7 (147.50)

**Table 2:** Isolation characteristics.

	Absolute numbers	Percentage of total cell number
Islets per animal	550	
Cells per islet	1872 ± 507	100%
Vitality (trypane blue)		>95%
Low fluorescent cells		20.03 ± 1.20%
High fluorescent cells		21.40 ± 10.8%

## ABBREVIATIONS

AUs: Arbitrary units
BSA: Bovine serum albumin
CMRL: Connaught Medical Research Laboratories medium
DMEM-F12: Dulbecco's Modified Eagles Medium
FAD: Flavin adenine dinucleotide
FCS: Fetal calf serum
FIUs: Fluorescence intensity units
KRH: Krebs-Ringer-HEPES
MEM: Minimal essential medium
NAD(P)H: Nicotinamide adenine dinucleotide (phosphate)
PI: Propidium iodide
PP: Pancreatic polypeptide
SEM: Standard error of the mean.
